# Intentional presence and the accompaniment of dying patients

**DOI:** 10.1007/s11019-023-10161-z

**Published:** 2023-06-20

**Authors:** Alexandra Guité-Verret, Mélanie Vachon, Dominique Girard

**Affiliations:** 1 Psychology Department, Université du Québec À Montréal, Montréal, Canada; 2 Réseau Québécois de Recherche en Soins Palliatifs Et de Fin de Vie, Québec, Canada; 3 Center for Research and Intervention On Suicide, Ethical Issues and End-of-Life Practices, Montréal, Canada; 4 Anesthesiology, Pain and Palliative Medicine Department, Radboud University Medical Center, Nijmegen, The Netherlands

**Keywords:** Presence, Accompaniment, End-of-life Care, Palliative care clinicians, Healthcare professionals, Dying, End-of-life, Hermeneutic phenomenology

## Abstract

In this paper, we offer a phenomenological and hermeneutical perspective on the presence of clinicians who care for the suffering and dying patients in the context of end-of-life care. Clinician presence is described as a way of (1) being present to the patient and to oneself, (2) being in the present moment, and (3) receiving and giving a presence (in the sense of a gift)*.* We discuss how presence is a way of restoring human beings’ relational and dialogical nature. To inform a different perspective on relational ethics, we also discuss how accompaniment refers to the clinician’s awareness of the human condition and its existential limits.

## Introduction

End-of-life care^1^ is emerging in diverse cultural settings around the globe. In many countries, access to end-of-life care, including preventing and relieving pain is now a human right (Boivin et al 2015; Downie et al. 2021; World Health Organization 2020). In parallel, Modern Western societies are characterized by difficulty giving meaning to end-of-life: death is socially fought and suffering is medically controlled and eliminated (Byock 2002; Lafontaine 2008). Uncertainties regarding life and death tend to be maximally reduced and the end of life became a part of a more secularized society, controlled by legislation and medical techniques. As a result, the phenomenon of end-of-life care has often been approached using these medical and legal lenses (Lafontaine 2008; Sallnow et al 2022; Vachon 2020). However, to capture the nuances of end-of-life care, it appears important to engage in interdisciplinary inquiry to explore and better understand this phenomenon, not limiting ourselves to legislative and medical lenses but also addressing the ethical and existential issues of caring for dying patients.

To accompany patients with humanity calls for a solicitude that differs from any duty of justice (Furstenberg 2021; Giblin 2002). In end-of-life care, accompaniment aims to restore a fundamental justice that passes through an ethical relationship between a patient and a clinician. As Quintin (2020) states, healthcare becomes an ethical act from the moment it is based on the ethics of human relationships—ethics brings humans back to their ontological interdependence, producing *connection* and *being*. However, is such ethics promoted today? Contemporary healthcare models fit into the framework of biomedical thinking and its positivist paradigm, concerned with capitalist efficiency, standardization, and control (Châtel 2016; Todres et al. 2007). In this context, it is frequent for clinicians to take care of patients by acting in a technical way (Châtel 2016). An assumption here is that it is difficult for clinicians to care for patients *while* accompanying them in their journey. It is arguably even more difficult for clinicians to understand what it means to accompany patients experiencing severe illness and approaching death because the work refers to a care whose scope is mainly ontological.

*To accompany* means *to be with* another person. Derived from Latin and anchored in the Christian tradition, the verb to accompany literally expresses *sharing bread with* and *going with*. It evokes the gesture of sharing something with a person by going metaphorically in the same direction as them. From this perspective, to accompany a person at the end of their life means engaging in a shared experience with the intention of being present and entering an encounter with them. In this sense, clinician presence plays a central role in accompaniment, and it appears clear that its aim goes beyond behavior and pertains to intentions.

If presence is recognized as one of the essential qualities of clinicians, there is no consensus on its definition in the literature or practice (Finfgeld-Connett 2008). Moreover, the current literature on clinician presence is mainly rooted in the field of nursing (e.g., Bright 2012; Du Plessis 2016; Fahlberg and Roush 2016; Vachon et al 2020). In contrast, a more general and existential conceptualization would be relevant, especially to inform thinking in medical humanities.

As we shall see, the meaning of clinician presence allows us to position accompaniment as an *art* rather than a technical practice (Châtel 2010). This ontological and epistemological position echoes the problematic position of healthcare concerning the “art of healing” discussed by Gadamer (1996). In his *Apologia for the art of healing*, Gadamer defended the idea of clinical practice as an art of hermeneutic relevance, requiring the exercise of presence and understanding. Among other philosophers, Gadamer suggested that illness, suffering, and death confront us ethically and force us to be present and understand. In this paper, we anchor our reflection on clinician presence in the hermeneutic phenomenological approach, from which we draw several existential concepts. Consistent with the art of healing, hermeneutic phenomenology is concerned with the idea that we must distance ourselves from scientific and everyday concerns to become aware of our ontological reality and gain a shared understanding of human experiences, such as the experience of the end of life.

This paper aims to provide an in-depth understanding of clinician presence in the context of end-of-life accompaniment. Drawing on the work of Châtel (2010), we will describe clinician presence as a way of (1) being present to oneself and the other, (2) being in the present moment, and (3) receiving and giving a presence (in the sense of a gift).These three themes allow us to play with the meaning of the word “present” as presence, present time, and gift.

This paper is divided into four parts. We first offer a brief introduction to palliative care and hermeneutic phenomenology. Secondly, we explore the three themes stated above. Thirdly, we address the risks and limits of clinician presence. We conclude with a case study.

Before going any further, we want to acknowledge the difficulty of being present with dying patients because of the organizational challenges in healthcare systems, dominated by concerns about efficiency pressures. The humanization of care that we call for does not rely solely on the work and responsibility of clinicians as individuals, but is part of a macrostructure that perpetuates problematic discourses and practices.

### Theoretical background

The following exposition of our theoretical background describes palliative care and hermeneutic phenomenology as distinct approaches before considering them together to shed light on the significance of clinician presence.

### Palliative care: An ethics of care

Although this paper discusses the presence of clinicians working in end-of-life care settings, we wish to focus first on the philosophy of palliative care as it is particularly relevant to understanding how and why to be present. Since its founding in the 1960s, palliative care has paved the way for humanistic care at the end of life. If, as Ariès (1975) said, death became a synonym for isolation in modern times, palliative care originates as a reaction to isolation. It offers a welcoming place where family, friends, and clinicians can co-construct, alongside the dying patient, the search for meaning in life and death. Different from curative medicine, palliative medicine is based on the idea that life should not be prolonged at all costs nor hastened. This idea nourishes an ethical reflection on the relationship between life and death (Vachon 2020).

The essential aims of palliative care are to alleviate the suffering of dying patients in the various dimensions of their experience (physical, psychological, social, existential, and spiritual) and to maintain their relationships (familial and social). Given this complex goal, palliative care is the responsibility of several clinicians from different backgrounds (e.g., physicians, nurses, humanistic psychologists). The concept of *total pain* is perhaps the most resonant to reflect how these clinicians try to understand the “whole overwhelming experience” of the suffering patient (Wood 2021). The philosophy of palliative care puts forward the possibility and relevance of combining technical expertise in pain relief with the ability to share the human experience of suffering. Palliative care, in this sense, calls for an ethics of care based on interdependence and reciprocity (Abel and Kellehear 2022; Vachon 2020). As we shall explore in the following sections, one’s presence allows one to see and meet the other as a subject similar to oneself.

### From phenomenology to hermeneutic phenomenology: The importance of intentionality and relationality in understanding a phenomenon

Phenomenology was founded by Husserl (1950, 1989) in the early twentieth century to overcome the limitations of the traditional scientific approach. Husserl argued that natural sciences had little to say about the human experience, its meaning, or its embedded relation to the world, even though our perception of objects and people primarily occurs through our relationship with them. As Husserl said, the consciousness is a consciousness *of* something. In other words, the consciousness does not exist by itself, it is directed towards objects and persons. In the work of Husserl (1950), this important idea refers to the notion of *intentionality* and contradicts the cartesian opposition between subject and object, and self and world. Indeed, we live in a relational mode, intentionally connected to the *life-world*, i.e., the world that supports us and provides us with the experiential basis of our acts. It follows that our knowledge begins with our experience and remains within the limits of our experience. However, Husserl stressed that we are generally unaware of our intentional acts, which prevents us from being conscious of what *is*. This problem refers to what Husserl called the *natural attitude*. The natural attitude would lead us to perceive the world as if it were an objective or impersonal place from which it was possible to detach ourselves. Husserl (1950) proposed a method—often referred to as a *reduction* or a *suspension of judgment*—to move from a natural attitude to a phenomenological one. Husserl’s method allows one to reconnect with the world because it involves bracketing or consciously suspending one’s preconceptions on a given phenomenon in favor of being available to the experience of this phenomenon. Intentionally directing one’s focus means shifting one’s attention to become aware of how the world gives itself to perception (Depraz et al. 2010).

Still concerned with the world or human experience as it is lived, Heidegger (1962) and Gadamer (1976) developed phenomenology by transforming it into a hermeneutic phenomenology because of the new place they give to interpretation and to the question of meaning (Grondin 2003). With Heidegger, Husserl’s focus on access to the consciousness’ content changed to a focus on the interpretation (the hermeneutic) of our relationship to the world we are always trying to understand and with which we exist. According to Heidegger, Husserl’s idea of suspension of judgment is limited because it does not reflect how our experiences are guided by our pre-understandings and existential conditions, i.e., our historically embedded ways of thinking. Three concepts of Heidegger’s work capture this new focus. (1) The being is a *being-there*. This concept unifies the present and the future, emphasizing the time and place in which we are. We should not conceive ourselves permanently or seek to be in the present moment but rather embrace our existential condition, which is that we become ourselves at each moment, projecting and opening ourselves toward diverse possibilities (projects, relationships, etc.). The fact that human existence is made of possibilities puts us, according to Heidegger, in a state of endless questioning, mainly because the utmost possibility is death. Time, therefore, reveals the ambiguity of human life: the future is both existence and finitude (de Beauvoir 1947). (2) The being is a *being-in-the-world*. Heidegger stated that we are not initially separated from the world but relate to it. The basis of human existence is not just how we know the world but also how we are (Laverty 2003). (3) The being is *thrown into the world*. This concept illustrates how pre-understanding defines the being in the world. Everyone is born into a pre-existing world that comes with preconceived understandings (e.g., interpretations, opinions, values, language) and given conditions (e.g., gender, nationality, body). In Heidegger’s opinion, this background represents an inherited tradition over which one has no control but must detach to unveil their conditions of existence (Grondin 2003). These three concepts are related to the idea of authenticity. According to Heidegger, the self is inauthentic when it is closed to conscience. In contrast, the authentic self can be revealed when we are aware of our own death as an omnipresent possibility and when we achieve a creative appropriation of our tradition. This appropriation, in turn, enables us to find a way of relating to others such that one is not lost in an everyday or instrumental mode (Wheeler 2011).

Gadamer (1976) did not call for a break from natural attitude (like Husserl did) and tradition (like Heidegger did). In his opinion, it is not possible, nor is it desirable, to understand a phenomenon without our prejudices being involved and to leave our tradition (Grondin 2003). Indeed, the work of hermeneutic phenomenology is a clarification of the conditions in which understanding takes place rather than a task aiming at unveiling or destroying our tradition. According to Gadamer, we come to an understanding of a phenomenon through dialogue, that is, through an *encounter* based on the *fusion* of two different traditions or *horizons* (Gadamer 1976), for example, the fusion of those of the clinician and the patient (Gadamer 1996). Through dialogue, one aims to encounter the other’s tradition from one’s tradition. It is precisely the awareness of one’s tradition and the recognition of the value of the other’s tradition that allows dialogue and leads to an understanding (Gadamer 1976). Gadamer also emphasized the linguistic nature of understanding, stressing that language invites us to recognize the limits of what can be known about something or someone. We access existence through words, narratives, and conversations, but our access to existence cannot be fully achieved. If language is unlimited, human expression is limited, thus there remains a gap between what is said and what is intended (Grondin 2003). Therefore, understanding is limited. Gadamer invites us to speak of understanding as an “event” of meaning. An encounter always opens up possibilities for further exploration, and it is agreed that the next encounter will reveal a new horizon.

In conclusion, hermeneutic phenomenology has given rise to existential and relational considerations that have profound implications for today’s standard conceptions of healthcare. Indeed, what is needed in end-of-life care encounters is a transformation of what Husserl called the natural attitude in order to achieve presence (for the clinician and the patient). But as Heidegger and Gadamer argued after Husserl, this transformation is limited. This means that presence is related to finitude, in the sense that clinicians can reach presence only if they keep in mind the finitude of human existence and the limits of human understanding. Also, presence is related to finitude because death directly calls for the deployment of presence (Vachon et al 2020). Indeed, a direct encounter with a dying person causes a rupture in the everyday experience and possibly in the natural attitude (Depraz et al. 2010). Part of our humanity “consists precisely in opening oneself to the death of the other,” according to Levinas (1999, cf. Sallnow et al 2022, p. 849).

### Ricoeur’s phenomenological and hermeneutical theory of suffering: Implications for end-of-life care and clinician presence

Ricœur (1994) provided a definition of suffering that allows us to link existential and relational considerations to end-of-life care encounters. Ricoeur (1990) is interested in human beings as “capable and suffering” actors, giving an account not just of our capacities (i.e., to do, to say, to tell, to estimate oneself as the other) but also of our fragilities. Drawing on this broad understanding, Ricoeur (1994) defined the experience of suffering as a) a decline in one’s capacity to act and b) an alteration of the relationship to oneself and to others. This definition reveals first of all the excessive nature of suffering, which does not affect an isolated part of the body but the way of being in the world (Marin 2013). It appears all the more true in the practice of end-of-life care, which focuses on the proliferation of pain in patients (Worms 2008). This definition also emphasizes that suffering impedes human intentionality and dialogue. Because it causes suffering, severe illness and the imminence of death make one less available to the world and thus trap one in a heightened self-awareness. No doubt that I am because I suffer, thought Ricoeur (1994). A poem by Rainer Maria Rilke, who suffered from an incurable disease, provides a telling example of this particular sense of existence: “Oh life, life, remaining always outside.” “So powerfully does pain cause us to withdraw from all external experience of the world and turn us back upon ourselves,” commented Gadamer (1996, p. 75). Suffering loosens the intentional threads that connect one to the world (Marin 2008). In other words, it narrows one’s horizon of possibilities and makes it difficult to project oneself into the future, projects, and relationships. The approach of death brings forward this narrowing of possibilities, emphasizing all the more the ultimate possibility: death. In this perspective, Ricoeur (1994) associated suffering with a crisis of meaning. The confrontation with suffering, illness and death triggers a search for meaning: action gives way to self-limitation vis à vis oneself and others, so what was taken for granted or normal now requires meaning. Recent research also suggests that suffering and serious illness represent a departure from one’s usual way of being, generating feelings of strangeness, absurdity, or unfamiliarity (Daneau et al. 2022; Hvidt 2017; Marin 2008; Svenaeus 2011; Van Lander 2020).

Ricoeur leads us to understand the experience of suffering as a reduction in the scope of intentionality and, consequently, an alteration of the capacities to act, relate and understand. We find this definition relevant to setting a realistic and humanizing goal of care in the context of end-of-life care. To alleviate the patient’s suffering at the end of life, the clinician may have the objective of increasing the patient’s intentionality, that is to say, restoring the patient’s relational capacity thus providing him or her with new opportunities for action, self-esteem and meaning. In our opinion, this is precisely the objective to which presence responds. But in order to achieve presence, the clinician must first be able to recognize the suffering patient. Ricoeur’s definition is also very useful in this regard. Indeed, because it is focused on existential themes that reveal something of the patient’s experience and the human condition, Ricoeur’s definition can help sharpen the clinician’s attention to the patient and therefore facilitate the recognition of the patient as a suffering human being.

### The meaning of clinician presence

In light of the theoretical context, the following section describes clinician presence as a way of (1) being present to oneself and the other, (2) being in the present moment, and (3) receiving and giving a presence (in the sense of a gift).

### Being present to oneself and the other

Presence is a way of being that refers to an awareness of the self, the other, and the relationship between self and other. In practice, one can develop such awareness by adopting a phenomenological attitude, as Husserl described it. Presence refers to the engagement that a person seeks to reach a mental and physical immobility to become responsive and inclusive. Presence precisely requires a change in the *direction* and the *quality* of attention (Depraz et al 2010). Indeed, presence stands in contrast with our tendency to a) spontaneously directs us towards an external and over-stimulating environment, and b) enjoins us to master and categorize everything (and everyone) rapidly (Depraz et al. 2010). Presence thus offers “a less means-oriented focus, where people and things are not quickly reduced to their use” (Todres et al. 2007, p. 54). To reach presence, one must also deepen the quality of their attention by bringing their cognitive activity inward. Presence refers to an ongoing movement of reception—to listen and “let things come”—in which one is conscious of their intentional acts and, therefore can be touched and be aware of being touched. Presence is *intentional* because it is non-directed, or directed toward a possible revelation (Depraz et al. 2010).

The non-directionality of presence explains why one’s presence opens the way to an encounter and a co-construction of meaning with another person. Presence may seem paradoxical because to reach others, one starts from oneself. Indeed, presence is known from within. A withdrawal from the world allows one to be present to oneself, which opens up the possibility of being present in the world and making the world present to oneself. From this perspective, one cannot be present without acknowledging human beings’ ambiguous and intersubjective condition, which is that situated subjects are never pure inwardness or pure externality (de Beauvoir 1947). As Ricoeur (1990) said, presence might be the fundamental nexus between being oneself and being in the world (cf. Bright 2012).

The non-directionality of presence also implies that presence is based on a tension between attention and inhibition. Tolerating this tension is not easy, just as tolerating human ambiguity is not easy. To be present, we must resist immediately filling the space that attention has freed up (Depraz et al. 2010). The tension between attention and inhibition is particularly confronting with regard to our preconceptions. Presence requires us to bracket our preconceived ideas and representations. In the context of end-of-life accompaniment, it requires a confrontation of our preconceptions of what a “good death” is, including questioning our sensitivities and values associated with suffering, vulnerable bodies, and death. In this regard, there is also an issue of social control over the patient that must be reflected upon (Depraz et al. 2010). To accompany is not to impose our preconceptions on a person or to guide a person on the assumption that we already know what they are experiencing and what is good for them (Châtel 2010). Nevertheless, hermeneutic phenomenology reminds us that we cannot completely abstract from our preconceptions, which means that the values and lived experiences of clinicians cannot be divorced from clinical practice. What is possible is to foster a self-reflective clinical practice, so we can make clear what we bring of ourselves and what we project into the care relationship.

In the context of end-of-life accompaniment, the tension between attention and inhibition is particularly confronting regarding our own finitude. The experience of the suffering patient and the context of care—which is explicitly related to death—call for a presence focused on the human condition. Heidegger (1962) insisted on that condition: the being is a *being-towards-death*. Ricoeur (1990) insisted for his part on the active *and* suffering nature of the being. Those philosophical stances are important to understand presence better. The recognition and awareness of finitude make one more attentive to the experience of the other and to one’s own experience about suffering (Bourgeois-Guérin and Beaudoin 2016). Ricoeur would say that because I am mortal, I can recognize myself in the dying patient’s suffering. He would also say that because I am mortal, the patient’s request for meaning (Why this disease? Why me?) challenges me personally. Perhaps human finitude is the most important meeting point with the patient, representing a shared vulnerability from which the clinician can reach presence and develop a reciprocal and ethical relationship with the patient. In *Oneself as another*, Ricoeur (1990) argued that we could more easily create an ethical movement between self and other by recognizing the universality of suffering and death.

Moreover, the recognition of one’s finitude might be important to take care for oneself as a clinician. In the context of end-of-life care, clinicians face continual exposure to intense suffering, which can be difficult to bear (Katz and Johnson 2016; Cross 2019; Rothschild and Rand 2006; Vachon and Guité-Verret 2020). Presence is a purposeful practice that might ease clinicians’ suffering in the face of human suffering, because presence is a response to suffering (Furstenberg 2021). Clinician presence might even be a way of surviving the powerlessness that comes with finitude.

### Being in the present moment

This section describes the space (here) and time (now) where the self and the other can meet. We refer here to presence as being in the present moment. The hermeneutic phenomenology approach invites us to live the present in the perspective of the past and the future. For example, there is a connection between presence and the dimensions of past and future when the clinician receives the patient’s story (which is part of the patient’s narrative identity) but also when the clinician is attentive to the history of his or her relationship with the patient, which includes an attention to the expansion of the clinical encounter. Thus, being in the present moment refers less to a fixed temporality than to an attention to the instability and the expansion of our experiences, which compels to be attentive to the ways in which the relationship with the patient changes (deepens) over time and with each encounter.

When clinicians have to be in the present moment, their work environment enjoins them to be concerned with the future and its prediction, taking them away from the present moment and its existential relationship to the continuity of time. The contemporary exigence of efficiency sticks us in a socially accelerated time and an over-stimulating environment that takes us away from existence (Rosa 2010). Hence, to achieve a non-directed presence, clinicians have to metaphorically get out of the ordinary time and space (Depraz et al. 2010; Vachon et al. 2020). Returning to the phenomenological attitude, we see that the change in our relationship to the world (i.e., in the direction and quality of our attention) includes a change in our relationship to time and space. Again, this change requires questioning one’s attitude and preconceptions, notably those associated with the modern paradigm of healthcare. This paradigm is characterized by a propensity *to do*—do more, all the time, more quickly (Todres et al. 2007). In the context of end-of-life care, this paradigm entails a propensity to do something in response to suffering and death, even though these existential realities require a way of being that is primarily intentional. The propensity to do, commonly viewed as the only and best way to respond to patients, is highly problematic as it encourages a rushed activation with suffering patients. Associating this modern propensity with a “natural attitude” can clarify how it pushes us to automatic gestures and removes us from our awareness of our intentions. In practice, adopting a natural attitude in the face of suffering and death often leads to more control and futile actions toward the patient, which also indicates a lack of connection with the patient’s experience and one’s own experience. Presence requires a shift from societal responses to death: “death and dying are increasingly controlled by heath systems” and intervention driven (Sallnow et al 2022, p. 853), which contrasts with the posture of *being with* the dying person. In order to adopt a phenomenological attitude, one should replace a common tendency to be active (rapid) with an unusual passivity (slowness) because only then could they receive something from the patient (an affect, a demand, a story, etc.) and be touched by it. As presence with the patient deepens, time seems to slow down and space seems to open up (Vinit 2016), fostering a connection to the lifeworld and to existence, making dialogue possible.

In parallel, the clinician must connect with the patient’s lived time, which is usually quite different from the accelerated time in which the clinicians work and live (Jacquemin 2005). The time related to end-of-life accompaniment is typically slow and fits particularly well with presence and, more importantly, is the ultimate time that the patient is living. Essentially, accompaniment depends on the capacity to know one’s rhythm and to feel the other’s. Being in the present moment is a process of resonance, a matter of affective attunement. “To accompany is first to respect the rhythm of the other without imposing one’s own. It is then to tune these two rhythms to enter, even fleetingly, into a common breath, into the singular rhythm of an always singular relationship”. (Châtel 2010, p. 5) It appears important to say that *resonance* is the human ability to be touched and directly concerns intentionality. In this regard, presence reconnects us to human resonance and releases us from an anesthetized relationship to the world.

### Receiving and giving a presence

According to the philosophy of palliative care, each person gives and transforms the other through a mutual gift of self (Châtel 2010), which takes up Gadamer’s idea of the fusion of horizons. Presence creates an affective relationship that differs from the usual vertical relationship in medicine, where the clinician gives and the patient takes. Presence can rebalance, through a movement of giving and receiving, the initial dissymmetrical position (and power) of the patient and the clinician (Furstenberg 2021). This dialectic of giving and receiving appears to be a fundamental aspect of the ethics of end-of-life care because it refers to a capacity for exchange. Dying patients may be experiencing great suffering, which reduces their capacity to act and enter into relationships, but the establishment of a reciprocal relationship enables them to give, which can alleviate their powerlessness (Ricœur 2007).

It is probably necessary to recall that reciprocity is not equality. As a matter of fact, a difference will persist between the patient and the clinician, particularly in terms of dependence, powerlessness, and suffering. Nevertheless, the clinician’s presence creates a relationship, a time, and a place through which the patient can give and receive, that is to be in contact, to dialogue, to tell a story, to transmit knowledge and values, etc. Living up to death, the dying patient is not a moribund and can act through the gift of self (de Lange 2014; Ricoeur 1990). In accompaniment, we argue that a dynamic of reciprocity is initiated when the clinician witnesses and inherits the work that the patient accomplishes in the face of suffering and death. The patient and the clinical relationship have something to teach us. For example, the patient helps us to adapt to death, which will inevitably come for him or her (soon) and us (later). The patient also teaches us that it is possible to connect despite the loneliness of suffering and that it is possible to suffer as much. These existential lessons or *gifts* remind us that the encounters with patients transform the clinicians and enable them to see new horizons (cf. Gadamer 1976).

The ability to give and receive is all the more important as it attests to human existence. Being is ruled by an economy of gifts that creates a bond between two persons (de Lange 2014), a bond that has strictly no value or meaning for the capitalist economy. Clinician presence feeds an ongoing movement by which the existence of the patient and the clinician are revealed, alongside the meaning of the experience of the end of life. In line with this perspective, accompaniment is a circular movement: the clinician who witnesses the patient’s life brings the patient into existence and, at the same time, the clinician discovers himself or herself as a witness, which brings him or her into existence and enables him or her to recognize and care for the patient (Quintin 2020).

### The risks and limits of clinician presence

There are limits to clinician presence, and these limits come with inherent risks. Châtel (2016) encourages us to use the metaphor of the tightrope walker to appreciate the role of clinicians who accompany dying patients. The point is that presence can be enriching and even comforting but can also be uncomfortable. To ignore this aspect would be to lose contact with its definition and purpose. The phenomenological attitude is risky because it brings us out of the mode of natural attitude and neutrality (Dufourmantelle 2011). By being present, clinicians return to their position as subjects, which brings them back to their finitude—not knowing, not having the solution, not doing, being vulnerable, etc. They confront their relationship to control and limits (Jacquemin 2002). They dare to simply be there without expectation (Châtel 2010). They risk the absence of reference points (Vachon 2014). They also take the risk of being emotionally moved by the radical otherness of the patient in front of whom they can only be there, unable to cure illness, eliminate suffering, or postpone death. Therefore, they leave the socially valued and often internalized posture of the omniscient and omnipotent (Robo 2021).

The risk of clinician presence also comes from the limited and fragile relationship with the patient; not only is the relationship between patient and clinician is limited by the end of life (the time left in the patient’s life), but also by the time limit that comes with the fast pace of the healthcare system (the clinician tries to find time to open and deepen the relationship). The clinician’s responsibility is to respond to the ethical injunction of being present to the other and cultivate their relationship despite its limits.

In addition, the clinical encounter and the meaning that emerges from it are neither continuous nor fixed once and for all. The encounter is a brief event of meaning (Gadamer 1976). The contact created must be maintained, deepen, and adjusted before it ends. In other words, the dialogue is infinite and expanding, and it is up to the individuals to end it when it feels right. For this reason, we should humbly speak of “moments of care” (Quintin 2020, p. 59). “Presence is so fleeting that we should, for the sake of caution and simplicity, speak only of moments of accompaniment and take care to detach ourselves from any other intention.” (Châtel 2010, p. 93). For the same reason, the clinician must leave the relationship when the time comes so that the contact does not become excessive or overstimulating for the patient. Hence, adapting to the patient at the end of life includes knowing when to withdraw, so the patient has access, after the contact, to a time of rest, passivity, and reverie (Van Lander 2020).

More broadly, the limits of the care relationship concern the limits of *suffering with* the other. The patient’s suffering may be unsubstitutable and unspeakable, yet the patient’s demand (for connection and meaning) is limitless (Ricoeur 1994). With this in mind, it is safer to accompany patients knowing the limits of our response to them, at the risk of suffering from increased powerlessness or compassion fatigue. To accompany does not mean carrying everything, caring for everything, or fulfilling all the patient’s needs. According to Jacquemin (2005), this refers to the limits of one’s responsibility and help in the context of end-of-life care, which is a less radical position than is, for example, Levinas’ (1990) doctrine of “infinite responsibility” towards the other. Despite the stated limits, the fact remains that entering and cultivating the relationship with the patient is the only ethical path.

Confronted with limits, clinicians may doubt their clinical competence, their way of being, and even the relevance of their presence with patients. In this regard, team discussions can help validate, discuss, transform, and overcome what seems to be healthcare impasses. For example, team discussions can encourage trust in the potentialities of the encounter despite its risks and limitations. Accompaniment aims to stay connected with limits (i.e., uncertainty, ambiguity, powerlessness, and finitude). *Being with* sometimes means *tolerating the suffering with*—creating a space for the patient’s suffering as much as one’s suffering. The aim of accompaniment is not to overcome limits, as it is taken for granted in Western societies, but to be personally shaped and challenged by the confrontation with limits (Denizeau 2019). An underlying quality of clinician presence is the capacity to name and contain human powerlessness.

### Case study: Mrs. K and the feeling of powerlessness

This concluding section illustrates the important issue of human powerlessness in end-of-life accompaniment through the case of Mrs. K, whom we have met before her death. Mrs. K was a fifty-year-old woman with advanced thyroid cancer. She had been admitted to palliative care after an acute respiratory distress syndrome followed by a tracheostomy. She was unable to communicate verbally due to the tracheostomy, so writing was the only way for the care team and her family to connect with her. Mrs. K’s relatives were very uncomfortable seeing her suffering and knowing that she was dying, especially since her tracheostomy. They were not often at the bedside, and when they were, they felt helpless and useless, which affected their ability to be present. Therefore, our role with Mrs. K was to offer a kind of presence that her relatives could not provide, due to their own suffering. Although the topic of this article is not the clinician’s meeting with the family, we can mention here that our role with the relatives was to help them better understand what they were experiencing and to validate their feelings of powerlessness.

The clinical encounter between Mrs. K and us was based on presence. First, Mrs. K initiated a relational movement with her demand for relationship and meaning—her demand took the form of apparent suffering, looks, cries, and written words/questions. Our role as clinicians was to recognize Mrs. K as a suffering person and to respond to her demand by cultivating presence, that is, by becoming present to ourselves and to her, by being in the present moment, and by allowing her to give (teach) us something. We invited Mrs. K to enter into presence with us, by inviting her to become more attentive to her own experience as well as to our relationship, which was an implicit way of encouraging her to adopt a phenomenological attitude. Our concern was to create the opportunity for Mr. K to awaken her consciousness and to enter into an authentic encounter, so that she could better understand what it was like to experience the end of her life, here and now. Over the course of two sessions, Mrs. K shared with us her fear of dying of respiratory failure. More deeply, she expressed anxiety about the reality of her decline and saw no meaning in life. We came to understand that severe illness had shattered her sense of identity, and created an existential crisis. She had always seen herself as an active woman, in control of her personal and professional life, and the end of her life appeared dramatically inconsistent with her life up to that point.

Before the third session, Mrs. K’s health condition deteriorated. Medical tests showed the development of brain metastases. Rapidly, Mrs. K lost the use of her right hand which affected her remaining ability to communicate with others. At the same time, Mrs. K also began to refuse medication. The therapeutic impasse seemed obvious at first, as it seemed impossible to put into words the patient’s suffering and alleviate it through medication or conversation. The silence of the dying patient was particularly difficult for the care team and the family to tolerate because it underscored their powerlessness. Faced with this situation, we had to focus even more on our intentionality and trust in the non-medical quality of presence. There was not much to *do*, but it was still possible to *be there with* her. So we set a new therapeutic goal: to be in contact with the powerlessness of the patient and our own powerlessness. We opened a dialogue in silence with Mrs. K. With her consent, we touched her hands and her hair. Over the days, we established what appeared to be a ritual: we sat at her bedside and stroked her hair, a gesture that led her to allow herself to cry (which she was not able to do otherwise in front of her family) and to fall asleep.

The case study suggests that a dialogue took place with Mrs. K (first through conversation, then, through touch and gaze) because we trusted the effect of presence. Because we intentionally focused our attention on the patient and, more broadly, on the human existential conditions we shared with her, we offered her the possibility of a meaningful dialogue, that is, a more active participation in life and a new meaning, at a time when action and meaning were precisely limited. Returning to Ricoeur’s (1994) definition of suffering as a difficulty to act and to relate, we can argue that our presence contributed to alleviating Mrs. K.’s suffering. We can also say that our presence eased our own suffering in the face of her impending death, because it allowed us to find a way to respond to her suffering. Through the enlivening and meaningful effects of presence, two people came to inhabit a relational world and find some comfort.

## Conclusion

This paper offers a phenomenological and hermeneutical perspective on the presence of clinicians who care for suffering and dying patients, using an original perspective on relational ethics in healthcare. This theoretical reflection helps to conceive how end-of-life accompaniment depends on the clinician’s experience of the human condition. Presence is a way of being and connecting with oneself and the other, restoring the relational and dialogical nature of human beings. The very condition of a patient-clinician encounter produces connection and meaning. Another contribution of this paper is to emphasize how the clinician can embody a particular presence to offer resonance in the face of suffering and death. If the vulnerability of the dying patient may be obvious, that of the clinician is often forgotten and disregarded. Therefore, this article contributes to filling a gap in the current literature on the clinicians’ work with patients. More broadly, accompanying patients on their way to death appears to be a way to respond in a more appropriate manner to the essential question of human suffering. It is through medical humanities that we may hope to find, on the one hand, a different kind of remedy to our modern relation to suffering and, on the other hand, a different response to patients’ suffering.

Although this paper emphasizes the ethical significance of accompaniment, it does not leave aside the limits of clinician presence. The suffering patient may have a difficult relationship with the world and others, and this existential situation can complicate the care relationship and even imply a loss of reciprocity. Being present appears even more difficult in today’s hyper-organized societies that tend to speed things up and push us toward insensitivity (Rosa 2019). Considering the reality of clinicians’ professional lives, which are characterized by the acceleration and hyper-medicalization of care, our description of presence may seem confrontational and somewhat idealistic. However, we believe that this does not preclude the need to distinguish between everyday/instrumental and authentic encounters to promote the humanity of end-of-life care and the dignity of the dying. The ethics of accompaniment requires us to be present and to cultivate a relationship through which the patient can reenter the cycle of human social life. Disease and death confront us ethically, forcing us to be present and to engage in dialogue. It is our hope that this article will open up a dialogue about clinician presence and contribute to the possibility of a more humanizing care environment for both patients and clinicians.

## Footnotes

1 End-of-life care aims at assisting people who are facing imminent or distant death and ensure quality of life until the end of their life. (Cf. Izumi et al. 2012) This broad end-of-life care definition overlaps with palliative and hospice care but could also include assisting a patient with a euthanasia or assisted suicide request.
